# Relationship between Muscle Function, Muscle Typology and Postural Performance According to Different Postural Conditions in Young and Older Adults

**DOI:** 10.3389/fphys.2017.00585

**Published:** 2017-08-15

**Authors:** Thierry Paillard

**Affiliations:** Movement, Balance, Performance and Health Laboratory, University of Pau and Pays de l'Adour Pau, France

**Keywords:** balance, postural control, postural performance, muscle strength, muscle power, muscle typology, postural condition, fall

## Abstract

Although motor output of the postural function clearly influences postural performance in young and older subjects, no relationship has been formally established between them. However, the relationship between lower-extremity muscle strength/power and postural performance is often pointed out, especially in older subjects. In fact, the influence of motor output may vary according to the postural condition considered (e.g., static, dynamic, challenging, disturbing). In static postural condition, there may be a relationship between lower-extremity muscle strength and postural performance when the value of muscle strength is below a certain threshold in older subjects. Above this threshold of muscle strength, this relationship may disappear. In dynamic postural condition, lower-extremity muscle power could facilitate compensatory postural actions, limiting induced body imbalance likely to generate falls in older subjects. In young subjects, there could be a relationship between very early rapid torque of the leg extensor muscles and postural performance. In the case of postural reaction to (external) perturbations, a high percentage of type II muscle fibers could be associated with the ability to react quickly to postural perturbations in young subjects, while it may enable a reduction in the risk of falls in older subjects. In practice, in older subjects, muscle strength and/or power training contributes to reducing the risk of falls, as well as slowing down the involution of muscle typology regarding type II muscle fibers.

## Introduction

In bipedal quiet stance, body balance involves keeping the vertical projection of the center of mass within the base of support. This situation corresponds to a static postural condition without deformation or displacement of the base of support. In turn, the deformation and/or displacement of the base of support corresponds to a dynamic postural condition which implies that the center of mass constantly remains above this base. In static condition, postural performance can be assimilated to the ability to minimize body sway (i.e., displacements of the center of gravity— or displacements of the whole body—and/or displacements of the center of foot pressure—or variation of the moment around the ankle) in conventional postural conditions (e.g., bipedal quiet stance), but it can also refer to the ability to maintain body balance in challenging postural conditions with small bases of support (e.g., a stance classed as a handstand, monopedal stance), thus avoiding falling or/and postural imbalance (Paillard and Noe, 2015). In dynamic condition, postural performance characterizes the ability to maintain body balance in disturbing postural conditions (e.g., displacement of pedal supports, displacement of the base of support), as well as in external mechanical disturbances (e.g., fast horizontal accelerations of the ground surface, unexpected percussion or pushing a large body segment requiring postural reactions) in order to avoid falling (Paillard and Noe, 2015).

Motor output of the postural function is closely related to lower-extremity muscle strength and/or power, and proves to be a determining factor in postural performance (Horlings et al., 2008; Melzer et al., 2009; Orr, 2010; Forte et al., 2014; Gomes et al., 2015; Han and Yang, 2015). The meta-analysis by Muehlbauer et al. (2015) showed correlations between lower-extremity muscle strength/power and postural performance in all kinds of subjects (i.e., children, adolescents and young, middle-aged, and old adults). In older subjects, the value of lower-extremity muscle strength and/or power contributes to accentuating or minimizing the risk of falls (Horlings et al., 2008). However, it is possible to decrease muscle strength without deteriorating postural performance, and conversely, an increase in muscle strength after a specific training program does not systematically result in an improvement of postural performance (Orr et al., 2008; Howe et al., 2011; Granacher et al., 2012; Muehlbauer et al., 2012). In fact, there would appear to be a threshold below which the lack of muscle strength degrades postural performance (Paillard, 2017). In turn, when the level of strength is beyond this threshold, an increase in lower-extremity muscle strength does not enhance postural performance (Paillard, 2017). Yet certain studies have shown that in healthy young subjects who present values of lower-extremity muscle strength well above this threshold, the strongest subjects display the best postural performance (Paillard, 2017). There is an ambiguity here that deserves to be clarified.

In fact, the postural condition under consideration (i.e., static or dynamic, more or less challenging or disturbing) probably influences the relationship between muscle strength/power, and postural performance in healthy young and older subjects, independently of the other factors likely to impact postural performance, such as sensory input, central integration, and control of the postural function. As motor output of the postural function is linked mainly to neuromuscular system efficiency, the structural and functional characteristics of antigravity muscles may also influence postural performance. The aim is therefore to propose mechanistic explanations of the relationship between motor output and postural performance in healthy young, middle-aged and older subjects through structural and functional considerations of the neuromuscular system in relation to various postural conditions.

## Muscle functional characteristics

The influence of lower-extremity muscle strength/power on postural performance was addressed in static and dynamic postural conditions.

## Static postural condition

**Figure 1:**
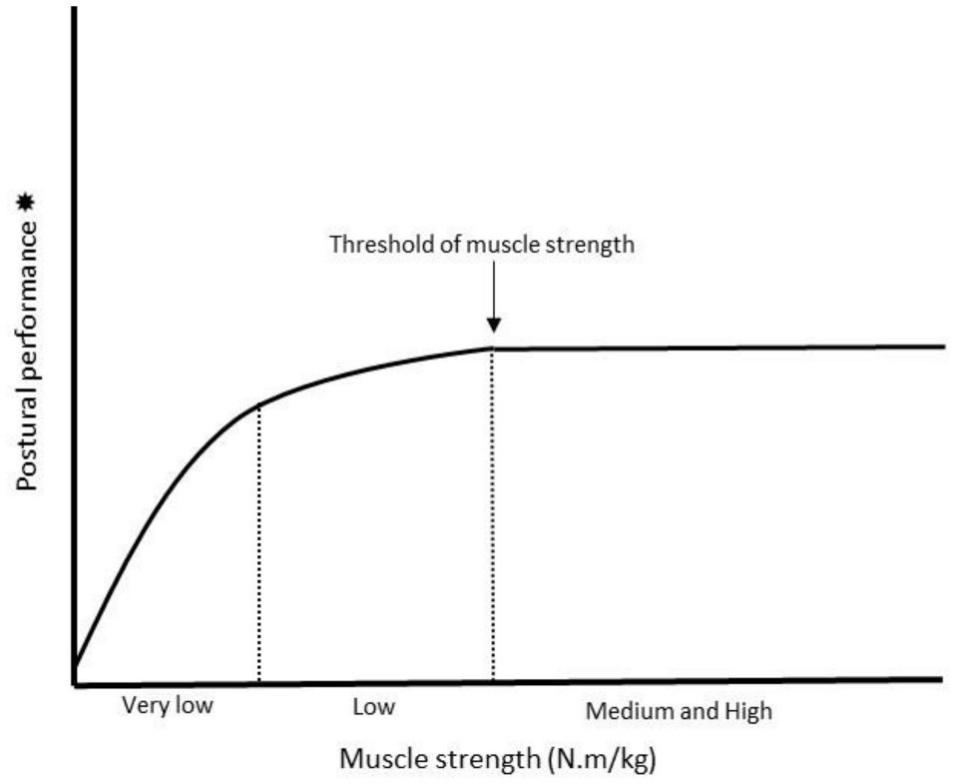
Illustration of the relationship between lower-extremity muscle strength and postural performance in static postural condition. An increase in muscle strength corresponds to an increase in postural performance until a certain threshold above which this relationship disappears (indicated by the arrow on the figure). Firstly, the curve would have a linear shape (i.e., for subjects presenting the lowest level of lower-extremity muscle strength i.e., “very low” zone on the figure) whereas secondly, it would tend to flatten (i.e., for subjects presenting a level of muscle strength slightly higher i.e., “low” zone on the figure). Thirdly, the curve would become horizontal (i.e., for subjects presenting the highest level of lower-extremity muscle strength i.e., “medium and high” zone on the figure). In fact, this curve would mean that the lower the level of muscle strength, the stronger the relationship between lower-extremity muscle strength and postural performance. ^*^When postural performance is evaluated through the recording of displacements of the center of foot pressure (COP), the level of performance is inversely proportional to the surface area (in mm^2^) of displacement of the COP i.e., the smaller the surface, the better the postural performance (Paillard and Noe, 2015).

Several factors are likely to have a negative impact on the relationship between lower-extremity muscle strength/power and postural performance in healthy older patients. With advancing age, intrinsic structural, and functional involutions in myotendinous and articular systems characterized by loss of muscle mass, weakening of contractility, a reduction in tendinous and muscle stiffness, the alteration of intermuscular coordination (agonist-antagonist muscle co-contraction) and a decrease in the range of motion of eversion-inversion of the foot (Vandervoort, 2002; Menz, 2015) inevitably contribute to lessening the motor output of the postural function (Bok et al., 2013; Cattagni et al., 2014; Menz, 2015). Based on these involutions, for a given postural condition, the effort required to ensure body balance necessarily increases (Skurvidas et al., 2012). Older subjects need more electromyographic activity (EMG) than young subjects to produce adequate torque to stabilize posture (Billot et al., 2010). Hence, it can be hypothesized that below a certain level of muscle strength, the increased EMG enabling the reduction in motor output related to age advancement to be offset is no longer possible and postural performance is altered negatively. This could explain why below a threshold of muscle strength, lower-extremity postural performance is reduced (Cattagni et al., 2014; Scott et al., 2014; Paillard, 2017). For instance, Cattagni et al. (2014) reported that below the threshold of 3.1 N·m·kg^−1^ for the plantar flexors, postural performance was dramatically diminished and body balance was even compromised. Scott et al. (2014) also clearly showed that subjects presenting the lowest quadriceps strength (<7 kg; lowest tertile) were those whose the risk of fall was the highest. In turn, when the force produced is above this threshold, an increase in lower-extremity muscle strength will engender no favorable consequence (or only a very slight one) for postural performance in a quiet upright position (i.e., static postural condition), whatever the subject's age (Paillard, 2017). An increase in lower-extremity muscle power after high-intensity interval training did not contribute either to enhancing postural performance in static condition in healthy sedentary old men (Sculthorpe et al., 2017). The existence of a relationship between lower-extremity muscle strength and postural performance in cases of weak muscle strength (i.e., below a certain threshold) in older subjects turns out to be highly probable in static condition (Figure 1). In this postural condition, an adequate level of muscle strength and tone (i.e., above this threshold) is enough to stabilize posture since there is no significant time constraint in postural regulation. In dynamic condition, the time constraint is greater and muscle strength value alone is not always sufficient to avoid body imbalance.

## Dynamic postural condition

Theoretically, muscle strength should be less determining than muscle power in dynamic postural condition, since the velocity of force expression should be fundamental when the pedal supports and/or the base of support are moving. In this case, the initiation of anybody imbalance must be counterbalanced as quickly as possible through opposing forces in order to overcome growing inertia as a function of time of unbalancing segmental movements and thus avoid falling. The decreased ability to develop force rapidly (i.e., reduced rate of force development) in older people could be associated with a lower capacity for neuromuscular response in controlling body balance (Izquierdo et al., 1999). Older fallers even demonstrated a reduced contractile rate of force development compared with non-fallers (Fleming et al., 1991). Conversely, lifelong football-trained older subjects showed superior rapid muscle force characteristics (elevated rate of force development and impulse, faster contraction times) and better postural performance compared with untrained age-matched individuals (Sundstrup et al., 2010). The retained capacity for rapid muscle force exertion and postural performance provides an enhanced ability to perform speed-demanding motor activities such as reversing a fall (Sundstrup et al., 2010).

In dynamic postural condition, such as gait for example, the useful force should be expressed as quickly as possible with an easing in articular mobility in order to resist a possible unbalancing postural shift. In case of postural imbalances, reactive actions to the perturbation must be sufficiently quick (i.e., 200–500 ms) to counteract a slipping limb and support body weight during reactive stepping (Cham and Redfern, 2001; Han and Yang, 2015). This requires force and velocity i.e., power (power is the product of force by velocity). Hence, the relationship between lower-extremity muscle power and postural performance could be particularly strong in dynamic postural conditions requiring compensatory postural actions. For the prevention of falls in older subjects, lower-extremity muscle power seems decisive in dynamic condition, while time reaction (both premotor and motor times) to postural perturbations also turns out to be of fundamental importance (Hong et al., 2014).

Most healthy and/or young subjects do not present any muscle strength deficit and are broadly above the threshold at which postural performance is diminished and balance compromised. Hence, only their ability to react quickly to postural perturbations should be taken into account. Palmer et al. (2015) showed that there is a relationship between very early rapid torque (0–50 ms) of the leg extensor muscles and postural performance in active college-aged females. However, there was no relationship between maximal strength nor late rapid torque (100–200 ms) and postural performance for this population. Only the expression of torque in the very first milliseconds would appear to be related to postural performance. Moreover, Johnson and Woollacott (2011) compared the postural response to fast horizontal platform perturbations in power-trained athletes (run 100 m in <11 s or a 40-m distance in <4.5 s and ongoing high-intensity training three times per week) and endurance-trained athletes (run 10 km under 31 min and ongoing running training of 60+ miles per week). Power-trained athletes responded with significantly shorter times to stabilize posture, shorter muscle-contraction onset times, and larger muscle response amplitudes (integrated EMG amplitudes) than endurance-trained athletes. These differences in postural and muscle response characteristics between power and endurance athletes were noted at higher perturbation velocities rather than at lower velocities. The difference in body mass cannot favor power-trained athletes more than endurance-trained athletes (84 vs. 66 kg) since from a biomechanical viewpoint, the heaviest subjects are also the worst in terms of postural performance (e.g., Olchowik et al., 2014). Regarding motor output of the postural function, evidence suggests that the ability to react quickly to postural perturbations in young subjects is based on the contractile qualities of the neuromuscular system which depend on its typological characteristics. It can therefore be wondered whether muscle typology is likely to influence (or not) postural performance in young and older subjects.

## Muscle typology

It is known that the size, recruitment threshold, contraction speed and force produced of type II muscle fibers (i.e., fast-twitch muscle fibers) are higher than those of type I muscle fibers (slow-twitch muscle fibers), but their fatigability is also greater (Henneman et al., 1965; Eberstein and Goodgold, 1968; Thorstensson et al., 1976; Herbison et al., 1982). Based on these data, both types of muscle fibers are likely to impact postural performance differently.

Miller et al. (2015) reported that in 30 healthy young subjects (24 ± 4 years old; height 174 ± 10 cm; weight 74 ± 16 kg), a higher percentage of type II fibers in the knee extensors (relative percentages of type I and II fibers determined indirectly) was associated with better reactive balance (latency time required to accommodate perturbations). These authors postulated that a relatively greater loss in the size of type II muscle fibers would reduce the individual's ability to produce force rapidly and perform functional tasks such as climbing stairs or avoiding obstacles quickly. On the contrary, in static postural condition, Jakobsen et al. (2011) observed a positive relationship in subjects aged 21–45 years between monopedal postural performance and the proportion of type I muscle fibers expressed as an area percentage. A large area percentage of slow-twitch muscle fibers may therefore affect postural performance positively. This result is also logical since the antigravity muscles that ensure body balance are mainly proximal and/or axial skeletal muscles which include essentially type I muscle fibers that are resistant to fatigue (Gurfinkel et al., 2006; Paillard, 2017). The discrepancy between the results of the previous two studies (Jakobsen et al., 2011; Miller et al., 2015) could be ascribed to the fact that a high percentage of type II fibers facilitates the ability to react quickly to postural perturbations, while a high percentage of I type fibers confers efficient quiet standing abilities in non-disturbed postural condition without causing early fatigue for long postural tasks and thus disturbing postural performance. This assumption would merit validation through further experimental work.

Regarding older subjects, Pajala et al. (2004) compared 97 monozygotic and 102 dizygotic female twins aged 64–76 years and inferred that the bodily structures and functions involved in postural regulation have high-to-moderate genetic influences. Indeed, some aged people may be genetically more disposed to poor postural performance than other people, placing them at increased risk of falls and mobility limitation. From a muscle typology point of view, type IIa and IIx muscle fibers decrease with age by area percentage, fiber number percentage, and mean fiber area, whereas type I fibers are clearly less affected in terms of area, number and size (Lee et al., 2006; Paillard, 2013). Lee et al. (2006) suggested that the modifications linked to type II fibers may affect muscle quality and thus contribute to disturbing postural performance. Sundstrup et al. (2010) also reported that lifelong football-trained subjects had a greater proportion of type IIa fibers and a smaller amount of type IIx fibers than age-matched untrained elderly subjects. A lower proportion of type IIx muscles fibers was observed in life-long endurance-trained and strength-trained master athletes, respectively, compared with age-matched untrained old subjects, while football training generated a downregulation in the proportion of type IIx fibers with an upregulated proportion of type IIa muscle fibers (Aagaard et al., 2007; Krustrup et al., 2010; Sundstrup et al., 2010). In fact, the lower proportion of fast fatigable muscle fibers (i.e., type IIx) observed in the lifelong-trained is likely to be functionally favorable, particularly in non-disturbed postural condition since muscle fatigue is likely to increase the risk of falls (Sundstrup et al., 2010). However, in situations where the ability to react quickly to postural perturbations is fundamental, lower type II fiber profiles are at greater risk of falls (Miller et al., 2015). In this context, Kramer et al. (2017) logically observed that older hip fracture patients showed extensive type II muscle fibers atrophy when compared with age-matched healthy subjects.

A high percentage of type II muscle fibers would appear to facilitate postural performance in young subjects and reduce the risk of falls in older subjects, especially in disturbing postural conditions, while a high percentage of type I muscle fibers may optimize postural performance over a long duration by limiting fatigue generating postural disturbance in both young and older subjects.

## Practical consequences

Independently of somatosensorial, balance (e.g., static, dynamic, challenging, disturbing conditions), flexibility and endurance sessions/exercises, older subjects should chronologically start intervention programs (fall risk prevention) aimed at developing lower-extremity muscle strength (at least triceps surae and quadriceps femoris). Once muscle strength has increased, they should pursue intervention programs by muscle power training then by reaction time training through different signals (visual and sound), and by dual-task (cognitive and postural tasks simultaneously) training. Even if it is possible to improve time reaction somewhat through specific training, this quality seems to be determined especially by muscle topology (i.e., neuromuscular qualities)—as part of motor output regardless of executive function—which is based mainly on genetic characteristics in both young and older subjects. Muscle strength and power training should also contribute to limiting the involution of muscle typology regarding type II muscle fibers in older subjects, while endurance training should reduce the fatigability likely to generate falls when standing upright over long periods.

## Conclusion

Evidence suggests that motor output influences postural performance in young and older subjects, but this influence would appear to differ according to the postural condition considered. In static postural condition, there seems to be a relationship between lower-extremity muscle strength and postural performance when the value of muscle strength is below a certain threshold in older subjects. Above this threshold of muscle strength, this relationship should disappear. In dynamic postural condition, lower-extremity muscle power appears to facilitate compensatory postural actions, limiting induced body imbalance likely to generate falls in older subjects. In young subjects, there is a relationship between very early rapid torque of the leg extensor muscles and postural performance. In the case of postural reaction to external perturbations, a high percentage of type II fibers seems to be associated with the ability to react quickly to postural perturbations in young subjects, while it should enable a reduction in the risk of falls in older subjects.

## Author contributions

The author confirms being the sole contributor of this work and approved it for publication.

### Conflict of interest statement

The author declares that the research was conducted in the absence of any commercial or financial relationships that could be construed as a potential conflict of interest.
